# Supplementary material for: Innovative RNAi Strategies and Tactics to Tackle Plum Pox Virus (PPV) Genome in Prunus domestica-Plum
Source: Plants (Basel). 2019 Dec 2;8(12):565. doi: 10.3390/plants8120565 (PMC6963518; doi:10.3390/plants8120565)

plants

Supplemental data to Figure 1

1. Cloning strategy of the amisiCPRNA constructs into pHellsgate

1.1. Subcloning in the pBluescript vector through Asp718 and HindIII

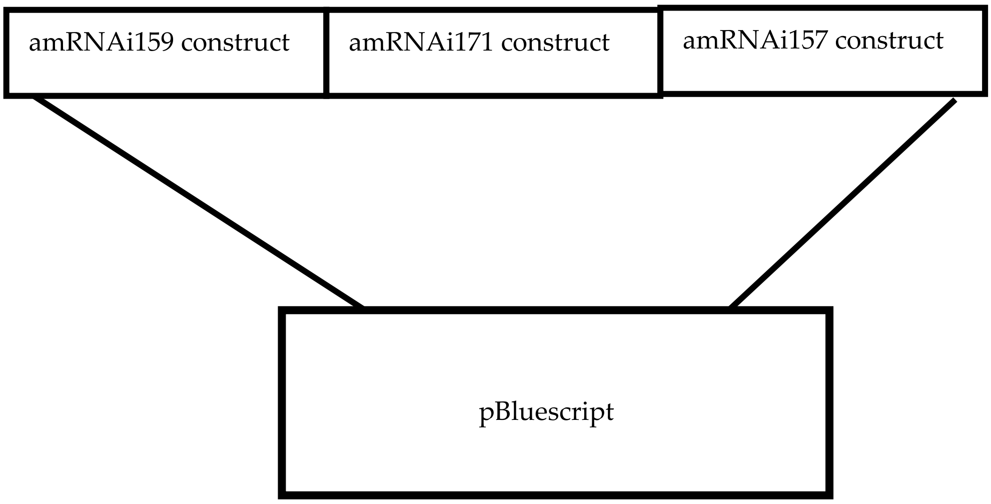

1.2. Cloning in the pHellsgate plant transformation vector

Excision of the amiRNA construct through Asp718+HindIII

Introduction of the recombinant insert in the pHellsgate vectors

(2a) The empty vector
(2b) The recombinant pHellsgate-B14

1.3. Cloning in the pHellsgate plant transformation vector double-digested with Asp718 and HindIII

amiCPRNA construct

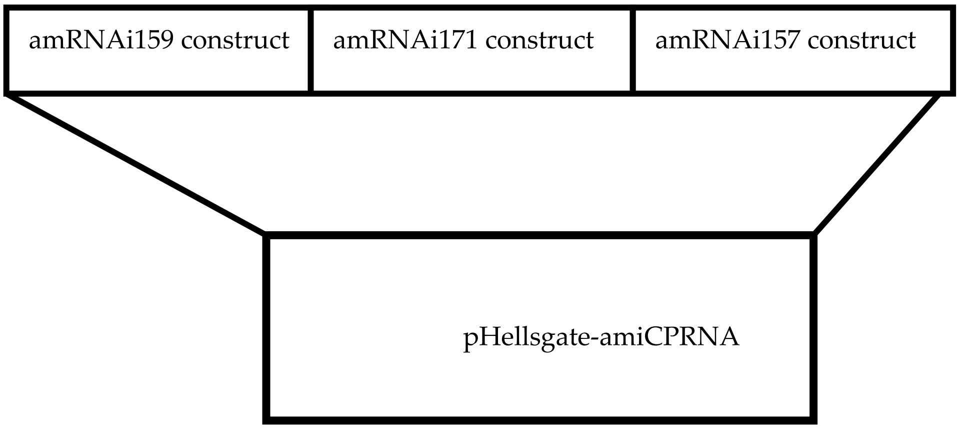

amisiCPRNA construct

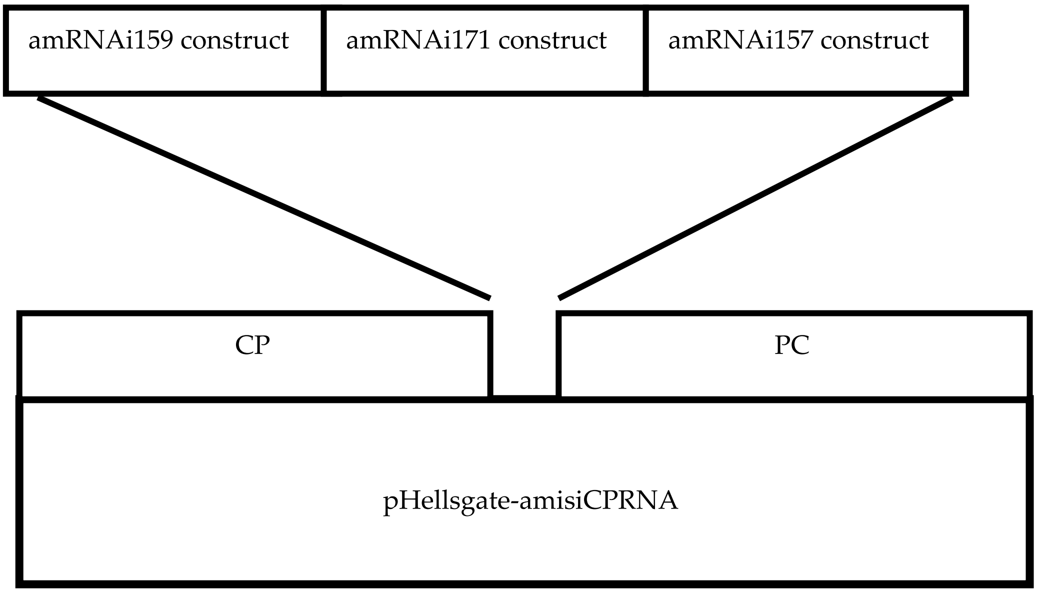

Supplement: Supplementary file 1 [file plants-08-00565-s001.pdf]
